# Supplementary material for: The practice of defensive medicine among Jordanian physicians: A cross sectional study
Source: PLoS One. 2023 Nov 9;18(11):e0289360. doi: 10.1371/journal.pone.0289360 (PMC10635536; doi:10.1371/journal.pone.0289360)
Supplement: S1 File — (PDF) [file pone.0289360.s001.pdf]

The practice of defensive medicine: a national survey of Jordanian physicians

Privacy Information

We inform you that your personal data, as reported in this survey, will be processed in a strictly confidential manner, and will be used only for scientific research purposes. Your personal data, as freely communicated by you, will be stored on electronic devices protected and processed confidentially for the goals of the present research and will not be passed to third parties. By filling in the information requested by this survey, you manifestly allow the processing of your personal data, as described above.
This research was approved by the Institutional Review Board (IRB) Committee at King Abdullah University Hospital (Ref number: 60/136/2020).

Part I. General questions on professional practice

**1. Gender**	
Male	Female

**2. Age (Enter a value between 18 and 100)**		
24-40	41-65	> 65

**3. In which region do you work? (Choose and option)**		
North	Middle	South

**4. How many years of professional experience do you have, <u>excluding training years</u>?**			
≤1	2-4	5-10	>10

**5. Specify you present working setting:**			
Government	Private	University-based	Military services

**6. Specify your specialty (Choose an option)**			
Anesthesia and resuscitation	Cardiology	Cardio-surgery	Dermatology
Dentist	E.R. Medicine	General surgery	General medicine and primary care
Gynecology	Internal medicine	Nephrology	Oncology
Orthopedics	Pathological anatomy	Pediatrics	Radiology
Specialty surgery	Urology	Other specialty: please specify	

**7. Where do you generally perform your activities? (Choose an option)**			
Outpatient	Consulting	E.R.	Department

**8. How many instrumental examinations and/or diagnostic tests and/or laboratory examinations do you perform on average in a typical working week?**

**9. How many patients do you follow/visit in a typical working week?**

Part II: Analysis of the professional behaviors in defensive medicine

Dear Colleague, in this section we ask you to answer some questions on your personal experience/ your opinion on the professional behaviors that can be attributable to the practice of defensive medicine in the medical profession, as far as the practice of defensive medicine is concerned.

Defensive medicine: "The prescription of medical procedures that are not strictly needed, patient selection, avoiding at risk procedures, requesting hospital admissions and specialty consulting or transfer when not strictly needed, etc. manly, although not exclusively, to reduce one's own exposure to the risk of legal claims." (U.S. Congress, 1994)

**10. During the last year, have you had the opportunity to adopt (or witness) defensive medicine behaviors? (Choose an option)**	
Yes	No

**11. In your opinion, what is the main cause of these behaviors of defensive medicine? (Rank from 0 to 10 where 0 is "the least frequent" and 10 "the most frequent")**	
**Mark**	**Question**
	Unfavorable legislation for the physician (MoH or JMA)
	Pressure from public and mass media opinion
	Risk of jeopardizing one's career and lose one's reputation or professional image
	Risk of being sued or incurring in legal issues for malpractice
	Risk of being asked for damage refunds
	Unbalancing of the doctor/patient relationship because of excessive pressure and expectations by the patients and/or his/her relatives
	Low trust in the management (Company, hospital, etc.)
	Low trust in insurance guidance and/or increase in insurance costs

 © All Rights Reserved

Dear Colleague, we remind you that your answers will be totally anonymous; you will be now asked to state your opinion on the frequency of various defensive medicine behaviors in your Organization, during a typical working week.

**12-17. In the following table, please quantify the frequency of the following behaviors prescribed/performed potentially in excess if compared to real needs**										
	**0%**	**1-10%**	**11-20%**	**21-30%**	**31-40%**	**41-50%**	**51-60%**	**61-70%**	**71-80%**	**81-90%**	**91-100%**
12. Specialty consulting/ referrals											
13. Laboratory exams with diagnostic goals											
14. Instrumental examinations and other diagnostic tests											
15. Prescribed drugs											
16. ER referrals/hospital admissions											
17. Transfers to other departments/hospitals											

**18-20. In the following table, please, quantify the frequency of the described situations:**											
	**0%**	**1-10%**	**11-20%**	**21-30%**	**31-40%**	**41-50%**	**51-60%**	**61-70%**	**71-80%**	**81-90%**	**91-100%**
18. The staff avoids to assist a patient with high risk of complications											
19. The staff avoids to perform potentially effective but high risk treatments or procedures.											

20. Requests for professional activities (visits, instrumental exams, etc.) by patients that can be interpreted as defensive medicine											

**21. The practice of defensive medicine is mainly: (Choose one option)**	
A factor favoring the professional practice	Go to Q 22
A factor limiting the professional practice	Go to Q 23
Not influent on the professional practice	Skip Questions 22 & 23

**22. State the reasons why the practice of defensive medicine can be considered a favoring factor for the professional practice (Rank from 0 to 10 where 0 is "the least frequent" and 10 "the most frequent")**	
It helps in medical decisions; it suggests the prescription of correct examinations and treatments.	
It improves the doctor-patient relationship/communications	
It is an aware act	
It satisfies the care needs of the patient	
It stimulates the medical activity and the working environment	
It protects against a possible professional responsibility	

**23. State the reasons why the practice of defensive medicine can be considered a limiting factor for the professional practice (Rank from 0 to 10 where 0 is "the least frequent" and 10 "the most frequent")**	
It conditions medical decisions	
It is an unaware act	
It forces to change medical activity or occupation	
It induces the performance of inappropriate examinations or treatments	
It limits the doctor-patient relationship/communications	

 © All Rights Reserved

It doesn't satisfy the care needs of the patient	
It doesn't protect against a possible professional responsibility	
It deprives the "professional ability" of its feature of being the main reference for the patients	

**24. Defensive medicine behaviors are, for the patients: (Choose one option)**	
A favoring factor	Go to Q 25
A limiting factor	Go to Q 26
Not relevant	Skip Questions 25 & 26

**25. State the reasons why the practice of defensive medicine can be considered a favoring factor for the patients (Rank from 0 to 10 where 0 is "the least frequent" and 10 "the most frequent")**	
It consolidates the central importance of the patient in performing medical acts	
It favors access to care	
It favors the perception of patient well-beings and satisfaction (increase in the perceived quality) It is an incentive to the assistance of patients who are more at risk	
It is an incentive to the assistance of patients who are more at risk	
It reduces patient risk (e.g. prescriptions of appropriate exams/treatments/interventions)	
It reduces waiting times	

**26. State the reasons why the practice of defensive can be considered a limiting factor for the patients (Rank from 0 to 10 where 0 is "the least frequent" and 10 "the most frequent")**	
It is a distraction from the main goal of medical acts, that is the "central importance of the patient"	
It increases the patients' risks (e.g. prescriptions of inappropriate and dangerous exams/treatments/invasive interventions)	
It increases waiting times	
It increases suffering (anxiety, stress, unsafety) and lack of satisfaction (decrease in perceived quality) It limits access to care	
It limits access to care	

 © All Rights Reserved

**27. Does the adoption of defensive medicine behaviors change the costs of healthcare interventions?**	
It increases the costs	Go to Q 28
It decreases the costs	Go to Q 29
It doesn ’t change the costs	Skip Questions 28 & 29

**28. In what percentage does the adoption of defensive medicine behaviors increase the costs of healthcare interventions? (Enter a value greater than 0%)**

**29. In what percentage does the adoption of defensive medicine behaviors decrease the costs of healthcare interventions? (Enter a value greater than 0%)**

**30. Does the adoption of defensive medicine behaviors change the likelihood of incurring in legal issues? (Choose one option)**	
It increases the likelihood of incurring in legal issues	Go to Q 31
It decreases the likelihood of incurring in legal issues	Go to Q 32
It doesn’t change the likelihood of incurring in legal issues	Skip Questions 31 & 32

**31. In what percentage does the adoption of defensive medicine behaviors increase the likelihood of incurring in legal issues? (Enter a value greater than 0)**

**32. In what percentage does the adoption of defensive medicine behaviors decrease the likelihood of incurring in legal issues? (Enter a value greater than 0)**

Part III: Actions aimed at changing defensive medicine behaviours

33. In your opinion, in the mid-term, are defensive medicine behaviors in medicine going to: (Choose one option)
Increase
Decrease
Remain unchanged

34. In your opinion, which personal actions might be effective in changing these defensive medicine behaviors? (Rank from 0 to 10 where 0 is "worst action" and 10 "the best action")	
Follow specific protocols and/or appropriate clinical evidence	
Continuously update knowledge, abilities and performance	
Appropriate multidisciplinary and multiprofessional communication	
Appropriate verbal and non-verbal communication with the patients	
Adequate healthcare documents and updated medical diary	
Participate to systematic and regular medical and clinical audits	
Strengthen one's own ethical and professional values	
Always report medical mistakes and participate to anonymous inquiries	

35. In your opinion, which external actions might be more effective in changing these defensive medicine behaviors? (Rank from 0 to 10 where 0 is "worst action" and 10 "the best action")	
Incentives and/or professional rewards for positive medical performances	
Greater interest of the public and mass media opinions in the healthcare activities that show value (not only in real/hypothetical malpractice)	
Greater support from the companies	
Greater guidance by insurance companies	
Reform of the regulations on professional responsibilities	

36. This is the end of the survey on defensive medicine. Would you like to add something else about the practice of defensive medicine?

 © All Rights Reserved
