# Supplementary material for: The practice of defensive medicine among Jordanian physicians: A cross sectional study
Source: PLoS One. 2023 Nov 9;18(11):e0289360. doi: 10.1371/journal.pone.0289360 (PMC10635536; doi:10.1371/journal.pone.0289360)
Supplement: S1 Table — (DOCX) [file pone.0289360.s002.docx]

**S2 Table: Frequency of main causes of DM behaviors among physicians by sector.**

|  | **Public hospitals** | | **Private hospitals** | |
| --- | --- | --- | --- | --- |
|  | Mean | Std. Deviation | Mean | Std. Deviation |
| **Unfavorable legislation for the physician (MoH or JMA)** | 6.038 | 3.212 | 5.611 | 2.818 |
| **Pressure from public and mass media opinion** | 6.288 | 3.372 | 4.832 | 2.801 |
| **Risk of jeopardizing one’s career and lose one’s reputation or professional image** | 5.525 | 3.307 | 4.968 | 2.680 |
| **Risk of being sued or incurring in legal issues for malpractice** | 5.250 | 3.286 | 5.179 | 2.939 |
| **Risk of being asked for damage refunds** | 5.300 | 3.328 | 4.968 | 2.792 |
| **Unbalancing of the doctor/patient relationship because of excessive pressure and expectations by the patients and/or his/her relatives** | 5.725 | 3.106 | 5.242 | 2.789 |
| **Low trust in the management (Company, hospital, etc.)** | 5.825 | 3.088 | 4.358 | 2.568 |

0 score indicates “the least frequent” and 10 score indicates “the most frequent”
