# Supplementary material for: The practice of defensive medicine among Jordanian physicians: A cross sectional study
Source: PLoS One. 2023 Nov 9;18(11):e0289360. doi: 10.1371/journal.pone.0289360 (PMC10635536; doi:10.1371/journal.pone.0289360)
Supplement: S2 Table — (DOCX) [file pone.0289360.s003.docx]

**S3 Table: Average frequency score of DM behaviors during a typical working week by sector.**

|  | **Public hospitals** | | **Private hospitals** | |
| --- | --- | --- | --- | --- |
|  | Mean | Std. Deviation | Mean | Std. Deviation |
| **Behaviors prescribed/performed potentially in excess if compared to real needs (assurance behaviors)** | | | | |
| Specialty consulting/ referrals | 3.663 | 3.138 | 2.489 | 2.322 |
| Laboratory exams with diagnostic goals | 4.425 | 3.467 | 3.379 | 2.772 |
| Instrumental examinations and other diagnostic tests | 4.313 | 3.433 | 2.747 | 2.593 |
| Prescribed drugs | 4.650 | 3.632 | 3.074 | 2.837 |
| ER referrals/hospital admissions | 3.354 | 3.527 | 2.421 | 2.426 |
| Transfers to other departments/hospitals | 3.413 | 3.415 | 2.232 | 2.238 |
| **Avoidance behaviors** | | | | |
| The staff avoids assisting a patient with high risk of complications | 3.688 | 2.954 | 3.266 | 2.713 |
| The staff avoids performing potentially effective but high-risk treatments or procedures. | 3.763 | 2.865 | 3.032 | 2.687 |
| Requests for professional activities (visits, instrumental exams, etc.) by patients that can be interpreted as defensive medicine | 4.463 | 3.218 | 2.937 | 2.418 |
