# Supplementary material for: The practice of defensive medicine among Jordanian physicians: A cross sectional study
Source: PLoS One. 2023 Nov 9;18(11):e0289360. doi: 10.1371/journal.pone.0289360 (PMC10635536; doi:10.1371/journal.pone.0289360)
Supplement: S3 Table — (DOCX) [file pone.0289360.s004.docx]

**S4 Table: Average frequency score of reasons why the practice of defensive medicine can be considered a favoring/limiting factor for the professional practice by sector.**

|  | **Public hospitals** | | **Private hospitals** | |
| --- | --- | --- | --- | --- |
| **Reasons why the practice of DM can be considered a favoring factor** | Mean | Std. Deviation | Mean | Std. Deviation |
| It helps in medical decisions; it suggests the prescription of correct examinations and treatments. | 6.176 | 3.340 | 6.778 | 2.547 |
| It improves the doctor-patient relationship/communications | 6.647 | 2.668 | 6.296 | 2.826 |
| It is an aware act | 6.000 | 2.598 | 6.222 | 2.953 |
| It satisfies the care needs of the patient | 7.000 | 2.716 | 6.519 | 2.276 |
| It stimulates the medical activity and the working environment | 5.471 | 2.695 | 6.481 | 2.343 |
| It protects against a possible professional responsibility | 6.706 | 2.995 | 6.889 | 2.665 |
| **Reasons why the practice of DM can be considered a limiting factor** |  |  |  |  |
| It conditions medical decisions | 5.907 | 3.217 | 6.089 | 2.704 |
| It is an unaware act | 4.574 | 3.106 | 5.444 | 3.034 |
| It forces to change medical activity or occupation | 5.315 | 3.267 | 5.089 | 2.670 |
| It induces the performance of inappropriate examinations or treatments | 5.944 | 3.037 | 6.044 | 2.402 |
| It limits the doctor-patient relationship/communications | 5.907 | 3.036 | 5.578 | 2.718 |
| It doesn ’t satisfy the care needs of the patient | 5.852 | 3.293 | 4.911 | 2.583 |
| It doesn't ’t protect against a possible professional responsibility | 5.926 | 3.302 | 5.200 | 2.642 |
| It deprives the “professional ability” of its feature of being the main reference for the patients | 6.815 | 3.192 | 6.600 | 2.359 |

0 is “the least frequent” and 10 “the most frequent”
