# Supplementary material for: The practice of defensive medicine among Jordanian physicians: A cross sectional study
Source: PLoS One. 2023 Nov 9;18(11):e0289360. doi: 10.1371/journal.pone.0289360 (PMC10635536; doi:10.1371/journal.pone.0289360)
Supplement: S4 Table — (DOCX) [file pone.0289360.s005.docx]

**S5 Table: Average frequency score of reasons why the practice of defensive medicine can be considered a favoring/limiting factor for the patients by sector.**

|  | **Public hospitals** | | **Private hospitals** | |
| --- | --- | --- | --- | --- |
| **Reasons why the practice of DM can be considered a favoring factor** | Mean | Std. Deviation | Mean | Std. Deviation |
| It consolidates the central importance of the patient in performing medical acts | 7.500 | 2.724 | 6.310 | 2.258 |
| It favors access to care | 7.386 | 2.461 | 6.524 | 2.039 |
| It favors the perception of patient well-beings and satisfaction (increase in the perceived quality) | 7.636 | 2.304 | 7.000 | 2.107 |
| It is an incentive to the assistance of patients who are more at risk | 6.364 | 3.073 | 6.500 | 2.200 |
| It reduces patient risk (e.g. prescriptions of appropriate exams/treatments/interventions) | 5.977 | 3.351 | 5.286 | 2.635 |
| It reduces waiting times | 5.318 | 3.442 | 4.952 | 2.996 |
| **Reasons why the practice of DM can be considered a limiting factor** |  |  |  |  |
| It is a distraction from the main goal of medical acts, that is the “central importance of the patient” | 4.966 | 3.145 | 6.833 | 2.666 |
| It increases the patients’ risks (e.g. prescriptions of inappropriate and dangerous exams/treatments/invasive interventions) | 4.828 | 3.526 | 6.533 | 2.488 |
| It increases waiting times | 5.069 | 3.770 | 7.500 | 1.757 |
| It increases suffering (anxiety, stress, unsafety) and lack of satisfaction (decrease in perceived quality) | 5.138 | 3.543 | 7.333 | 2.155 |
| It limits access to care | 4.310 | 3.371 | 6.233 | 2.956 |

0 is “the least frequent” and 10 “the most frequent”
