# Supplementary material for: The practice of defensive medicine among Jordanian physicians: A cross sectional study
Source: PLoS One. 2023 Nov 9;18(11):e0289360. doi: 10.1371/journal.pone.0289360 (PMC10635536; doi:10.1371/journal.pone.0289360)
Supplement: S5 Table — (DOCX) [file pone.0289360.s006.docx]

**S6 Table: Average effectiveness score of strategies in changing defensive medicine behaviors by sector.**

|  | **Public hospitals** | | **Private hospitals** | |
| --- | --- | --- | --- | --- |
|  | Mean | Std. Deviation | Mean | Std. Deviation |
| **Personal actions** | | | | |
| Follow specific protocols and/or appropriate clinical evidence | 8.063 | 2.538 | 8.537 | 2.041 |
| Continuously update knowledge, abilities, and performance | 7.913 | 2.615 | 8.789 | 1.700 |
| Appropriate multidisciplinary and multi-professional communication | 8.038 | 2.236 | 8.526 | 1.675 |
| Appropriate verbal and non-verbal communication with the patients | 7.625 | 2.567 | 8.074 | 2.069 |
| Adequate healthcare documents and updated medical diary | 7.788 | 2.390 | 8.179 | 2.222 |
| Participate to systematic and regular medical and clinical audits | 7.663 | 2.439 | 7.474 | 2.633 |
| Strengthen one’s own ethical and professional values | 7.613 | 2.674 | 8.358 | 1.962 |
| Always report medical mistakes and participate to anonymous inquiries | 7.100 | 2.722 | 7.663 | 2.716 |
| **External actions** | | | | |
| Incentives and/or professional rewards for positive medical performances | 7.063 | 3.016 | 7.200 | 2.612 |
| Greater interest of the public and mass media opinions in the healthcare activities that show value (not only in real/hypothetical malpractice) | 6.938 | 2.914 | 7.158 | 2.679 |
| Greater support from the companies | 6.563 | 2.823 | 6.337 | 2.797 |
| Greater guidance by insurance companies | 5.938 | 3.131 | 6.021 | 2.932 |
| Reform of the regulations on professional responsibilities | 7.263 | 2.704 | 7.347 | 2.763 |
